# Interpretable Composition-to-Curve Prediction of Payne-like Softening in an Unvulcanized BR/BR–VMQ Benchmark: Critical-Strain Scaling and Qualitative Molecular-Dynamics Context

**DOI:** 10.3390/polym18141761

**Published:** 2026-07-18

**Authors:** Yancai Sun, Feng Shi, Jian Xu, Wenjuan Bai, Dianming Chu, Peiwu Hou, Wenzhong Deng

**Affiliations:** 1 College of Mechanical and Electrical Engineering, Guilin University of Aerospace Technology, Guilin 541004, China; 2024025@guat.edu.cn; 2 College of Mechanical and Electrical Engineering, Qingdao University of Science and Technology, Qingdao 266061, China; bwj@qust.edu.cn (W.B.); chudianming@qust.edu.cn (D.C.); 4025037014@mails.qust.edu.cn (P.H.); 3 University Engineering Research Center of Non-Standard Intelligent Equipment and Process Control Technology, Guilin 541004, China; 4 Guangxi Engineering Research Center for Advanced Process Manufacturing of Precious Metal New Materials, Guilin 541004, China; 5 Academy of Aerospace Solid Propulsion Technology, Xi’an 710025, China; shifengcqu@126.com; 6 Guilin Rubber Industry R&D Institute Co., Ltd., Guilin 541004, China; xujian7@cncec.cn

**Keywords:** Payne effect, filled elastomer, composition-to-curve prediction, critical-strain scaling, molecular dynamics, bound rubber

## Abstract

Predicting a filled elastomer’s strain-amplitude softening curve from formulation descriptors is difficult because the response combines matrix softening, filler-network breakdown, and interfacial effects. We reanalyze a public benchmark of 47 unvulcanized butadiene-rubber (BR) and butadiene-rubber/silicone-blend (BR–VMQ) compounds (carbon black, precipitated silica, and nano-CaCO_3_; 5 to 30 phr) to test an interpretable composition-to-curve model. A per-compound Kraus-type critical strain $\gamma_{c}$ first overlaps the normalized total-softening curves onto a shared shape ($R^{2}=0.98$; a necessary but not sufficient precondition for universality). A structural-kinetics model—a machine-learned parameterization of a Kraus-type form, not a new constitutive law—then predicts the full curve from the rubber family and loading (root-mean-square error (RMSE) of 0.031), with a held-out Payne-like-drop mean absolute error (MAE) of 0.040, tied with a random forest (p=0.87) and matched by a full-curve black-box baseline; the Kraus form thus buys interpretability at no cost to accuracy. Because the compounds are unvulcanized, the target is the total softening—matrix-dominated for the blend—not an isolated Payne term. Explicit filler-chemistry labels and coarse-grained molecular-dynamics descriptors add no held-out value beyond family and loading, so the molecular dynamics is used as qualitative structural context rather than for quantitative mechanism validation; this bounds the claim to the present benchmark and does not imply that filler chemistry is unimportant in general.

## 1. Introduction

Reinforcing fillers raise the modulus of an elastomer by one to two orders of magnitude, far beyond the hydrodynamic (Einstein–Smallwood) contribution of rigid inclusions [1,2,3]. The excess stiffness originates in a percolating filler network and in the layer of polymer immobilized at the filler surface (“bound rubber”). Both are fragile under deformation: as the strain amplitude grows, the storage modulus $G^{\prime }$ decreases reversibly from a low-strain plateau $G_{0}^{\prime }$ to a high-strain plateau $G_{\infty }^{\prime }$. This amplitude dependence is the Payne effect [4,5,6], and its magnitude—conveniently summarized by the drop fraction $\Delta =\left(G_{0}^{\prime }-G_{\infty }^{\prime }\right)/G_{0}^{\prime }$—is among the most consequential design variables in rubber compounding, governing the rolling resistance, heat build-up, and dynamic stiffness of tires, seals, and vibration mounts.

Two pictures, not mutually exclusive, dominate the interpretation of Payne-like softening. In the filler-network picture, the modulus drop reflects the progressive rupture of filler–filler contacts and agglomerates [1,6,7]. This process is commonly represented by a strain-driven deagglomeration/reagglomeration balance and the widely used Kraus-type empirical law

(1) $$G^{\prime }\left(\gamma \right)=G_{\infty }^{\prime }+\frac{G_{0}^{\prime }-G_{\infty }^{\prime }}{1+{\left(\gamma /\gamma_{c}\right)}^{2m}},$$

where $\gamma_{c}$ is the critical strain and *m* is an exponent. In the bound-rubber picture, the modulus drop reflects changes in immobilized chains and polymer–filler contacts, an interpretation connected to the glass-transition gradient near the filler surface [1,8,9]. Cluster-aggregation theories [10,11,12] connect the network elasticity to the fractal structure of the filler clusters, with interfacial physico-chemistry governing dispersion and flocculation [9], and continuum strain-amplification models capturing the nonlinearity [13]. All of these reproduce a measured curve, but their parameters are extracted *per material*: they summarize an experiment; they do not predict a new compound from its composition or its interfacial physics.

A complementary and very active line of work infers *linear* viscoelastic relaxation spectra from dynamic data—by Tikhonov regularization, Prony/relaxation-spectrum methods [14], or, recently, neural spectral inversion—and these are often cast in Cole–Cole or generalized-Maxwell forms fitted to dynamic mechanical analysis (DMA). However, a linear spectrum is, by definition, independent of strain amplitude; it returns the same $G^{\prime }$ at γ=0.1% and at γ=100% and therefore predicts *zero* Payne softening. The Payne effect lies precisely in the strain-amplitude dependence that linear inversion discards. This is not a deficiency of any particular spectrum estimator—it is a structural limitation of the linear-response framework itself.

Rheology- and physics-informed neural networks have made data-driven constitutive modeling of complex fluids practical [15,16,17,18,19]. For elastomers specifically, interpretable and physics-augmented architectures now recover physically named constitutive parameters: constitutive artificial neural networks (CANNs) and their physics-enforcing variants learn hyperelastic laws whose weights are moduli and stiffness coefficients [20,21], while viscoelastic and thermodynamics-based extensions—vCANNs, thermodynamics-based networks, and neural-ODE viscoelasticity—keep the relaxation spectrum interpretable under free-energy and dissipation constraints [22,23,24]. A recent review surveys this physics-informed effort in polymers [25]. In parallel, data-driven models map rubber compound recipes to scalar properties [26,27,28]. Three gaps remain for filled-rubber amplitude softening. These interpretable viscoelastic learners target rate- and relaxation-dependent (time- and frequency-domain) nonlinearity rather than the strain-amplitude softening that defines the Payne effect; the recipe-to-property regressors return isolated numbers rather than an interpretable modulus–strain curve; and the mechanistic Payne models that do capture amplitude softening are not composition-aware [29]. The sharper formulation question for filled-rubber amplitude softening is therefore whether a compact, interpretable model can estimate the entire measured curve from formulation descriptors while preserving physically named curve parameters. Per-compound Payne fits answer how a measured curve can be summarized; they do not by themselves answer whether a new compound can be predicted from its composition.

**Figure 1:**
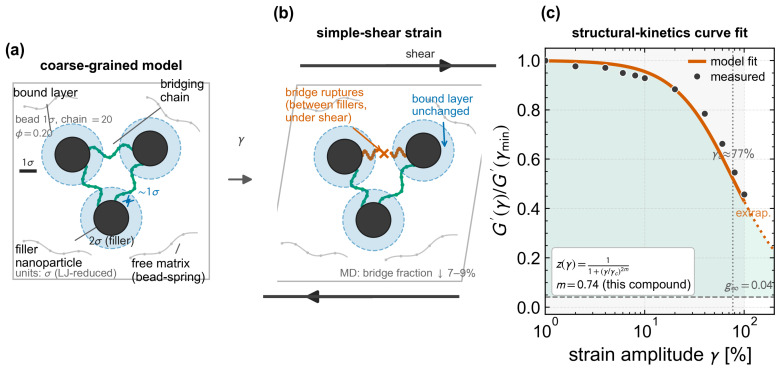
Concept of the structural-kinetics model. (**a**) Coarse-grained molecular-dynamics (MD) model (ϕ=0.20, below filler percolation): filler particles (diameter 2σ in Lennard-Jones reduced units, where the polymer bead diameter is σ) wrapped in a bound polymer layer (beads within 2σ of a filler centre) and linked by bridging chains of 20 beads in a free matrix; lengths are given in reduced units, and the schematic is not to physical scale. (**b**) Under *simple-shear* strain (horizontal shear arrows), a *fraction* of the interparticle bridges rupture—in the MD simulations, the bridge fraction falls by only 7–9%, while the bound fraction is essentially unchanged (<1%)—reducing network elasticity (the Payne mechanism); the depicted bond scission is shear-induced, not tensile failure. (**c**) A representative *measured* Payne-like curve (points; BR–VMQ/CaCO_3_, 5 phr—the compound nearest the median drop) with its structural-kinetics fit z(γ) (line; $g_{\infty }=0.04$, $\gamma_{c}=77\%$, m=0.74): solid over the measured 1–100% window, dotted in extrapolation to the residual plateau. Panels (**a**,**b**) are schematic.

The practical goal of this study is to predict the whole amplitude-softening curve of a filled compound directly from its formulation, so that the dynamic stiffness loss of a new recipe—the property that governs rolling resistance and heat build-up in the finished part—can be anticipated from the rubber family and filler loading without first running the strain sweep. We therefore treat the post-fit critical-strain overlap of the measured curves (Section 3.2) as an empirical starting point, not as the central result. The main test is composition-to-curve prediction: a structural-kinetics model predicts the parameters of a bounded Payne-like curve from the rubber family and filler loading, so that the output stays a full, interpretable $G^{\prime }\left(\gamma \right)$ response rather than a single scalar drop (Figure 1). Two supporting analyses frame this prediction. The dynamic mechanical analysis (DMA) subset checks whether a linear viscoelastic descriptor relates to the fitted scaling strain, and coarse-grained molecular dynamics (MD) supplies qualitative structural context—bound-layer screening and bridge rupture—while feature ablations test whether MD descriptors or explicit filler-chemistry labels add any predictive information. Because the compounds are unvulcanized, the target is the total measured softening, which includes a raw-rubber matrix contribution, not an isolated filler-network Payne term.

## 2. Data and Methods

### 2.1. Public Cross-Filler Benchmark

This work reanalyzes the openly licensed (CC BY 4.0) “Mars rubber” dataset [30,31]. It was chosen because it is, to the best of our knowledge, the only public dataset that measures the Payne effect across a consistent cross-chemistry, cross-loading matrix of compounds under a single protocol; its open CC BY 4.0 licence permits redistribution and makes the entire analysis an openly reproducible benchmark, which a proprietary in-house dataset could not. It contains strain-amplitude sweeps of the storage shear modulus (the Payne measurement; MonTech D-RPA3000 rubber process analyzer (MonTech, Buchen, Germany), 1 Hz, 100 °C, strain 1 to 100%) for 47 compounds, and temperature-sweep DMA (Gabo Eplexor 2500; GABO Qualimeter, Ahlden, Germany; 10 Hz, 2 K/$\min^{-1}$) for 17 of them (16 of which also have a usable paired Payne-like drop, used for the DMA correlation below). The compounds are butadiene rubber (BR) and BR/silicone (BR–VMQ) blends, each filled with three carbon blacks (N220, N330, N550), three precipitated silicas (Zeosil 1115, Ultrasil 7000, Ultrasil 9100), and nano-CaCO_3_ at 5, 15, and 30 phr, plus unfilled references—a genuine cross-chemistry, cross-loading matrix (25 BR + 22 BR–VMQ).

**Figure 2:**
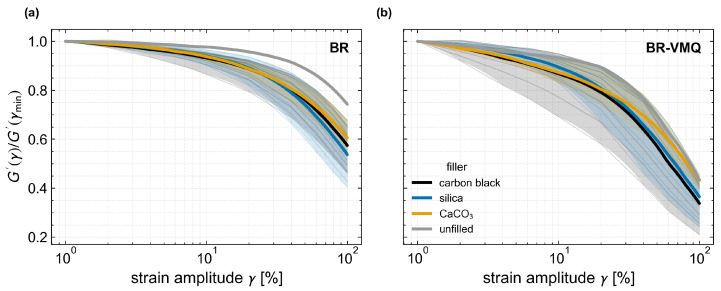
Normalized cross-filler Payne-like curves $G^{\prime }\left(\gamma \right)/G^{\prime }\left(\gamma_{\min}\right)$, colored by filler class, for BR (**a**) and BR–VMQ (**b**) compounds. Strain-amplitude sweeps were measured at 1 Hz, 100 °C, and 1 to 100% strain for 47 compounds. Active fillers (carbon black, silica) produce the most softening; nano-CaCO_3_ produces the least.

The raw semicolon-delimited tables were converted into a compound-level analysis table containing the material identity, rubber family, filler class, loading, measurement protocol, strain amplitude, and storage-modulus response. For each compound, the derived quantities include the drop fraction Δ, the half-softening strain, the tanδ peak, and the glassy/rubbery moduli. The processed data were checked against expected material behavior: the unfilled BR reference shows a glassy-to-rubbery storage-modulus ratio of $∼\;10^{3}$, with a tanδ peak at −93 °C (the BR glass transition), and the measured drop increases with loading and with filler activity (Figure 2).

### 2.2. Baselines

Three baselines bound the problem: (i) a *linear viscoelastic spectrum* predicts no amplitude softening; its error on Δ is the entire observed drop; (ii) the *Kraus* law, Equation (1), fitted for each compound (all 47 fits in Table S1), is the gold-standard empirical description of a single curve; and (iii) a *ridge regression* predicts the scalar Δ from composition features (family, filler-class one-hot, log loading), representing a strong scalar predictor with no curve and no mechanism.

### 2.3. Structural-Kinetics Model

A structural-integrity variable z(t)∈[0,1] is introduced with breakdown/reformation kinetics (Equation (2)):

(2) $$\frac{dz}{dt}=-\;k_{d}\left(\gamma \right)\;z\;+\;k_{r}\;\left(1-z\right),$$

where $k_{d}$ is the strain-driven breakdown rate and $k_{r}$ is the thermal reformation rate. The variable *z* is phenomenological: it represents the intact fraction of the strain-labile filler network rather than a directly measured microstructural quantity. The coarse-grained molecular dynamics of Section 3.5 reproduce the corresponding structural change—rupture of interparticle bridges while the bound layer remains essentially intact—and is offered as qualitative support for this picture rather than as a calibration or a measurement of *z*. Under a steady amplitude sweep, this relaxes to a sigmoidal steady state in logγ; the corresponding closed form is adopted:

(3) $$z\left(\gamma \right)=\frac{1}{1+{\left(\gamma /\gamma_{c}\right)}^{2m}},\;\frac{G^{\prime }\left(\gamma \right)}{G^{\prime }\left(\gamma_{\min}\right)}=g_{\infty }+\left(1-g_{\infty }\right)\;z\left(\gamma \right),$$

where the residual-plateau fraction is $g_{\infty }=G_{\infty }^{\prime }/G_{0}^{\prime }$, $\gamma_{c}$ is the critical strain, and *m* is the breadth. Equation (3) shares its functional form with the Kraus law (Equation (1)); the model is therefore a machine-learned parameterization of a Kraus-type form, not a new Payne-effect constitutive law. The essential difference from Kraus is that the parameters $\left(g_{\infty },\gamma_{c},m\right)$ are not fitted per sample but are the output of a small multilayer perceptron (MLP—a statistical regressor, to be distinguished from the physical filler network of Equation (1)) with two hidden layers of 24 units and SiLU activations acting on compound features, with $g_{\infty }=\sigma \left(\;\cdot \;\right)$, $\gamma_{c}\in \left(0.5,200\right)\%$ and m∈(0.2,2.0) enforced by bounded activations to keep the curve physical. The network minimizes the mean squared error of the full normalized curve $G^{\prime }\left(\gamma \right)/G^{\prime }\left(\gamma_{\min}\right)$ over all strain points (Adam, 4000 steps, weight decay $10^{-4}$). Because the parameters are predicted rather than fitted, the model generalizes to a held-out compound; because they are the parameters of Equation (3), every prediction is a bounded curve with physically named parameters.

Three feature sets were compared. The *minimal* set uses only the rubber family and log loading. The *composition* set adds the filler-class one-hot; the *MD-transfer* set instead adds the MD structural descriptors of Section 2.4. The latter two test whether explicit filler-chemistry identity helps at all—the ablation of Section 3.8; the per-filler-class breakdown of these held-out errors is given in Table S2. For MD-transfer, each compound is assigned the descriptors of the MD cell whose polymer–filler interaction matches its filler chemistry, using a physically motivated ranking of reinforcing activity: nano-CaCO_3_ (inactive) to weak ($\varepsilon_{pf}=0.5$), precipitated silica to intermediate ($\varepsilon_{pf}=1.0$), and carbon black (most reinforcing) to strong ($\varepsilon_{pf}=2.0$); the unfilled reference carries zero descriptors. This activity ranking—carbon black > silica > CaCO_3_—is textbook reinforcement knowledge [1,7], fixed independently of the benchmark; the MD columns are *z*-scored. The mapping is a proxy rather than a calibration (Section 3.8 and Section 4).

### 2.4. Coarse-Grained Molecular Dynamics

Coarse-grained molecular dynamics is an established route to the structure and mechanics of filled polymers [32,33]. A Kremer–Grest bead–spring melt was simulated [34] in LAMMPS (version 22 Jul 2025) [35,36], in Lennard-Jones (LJ) reduced units; all simulation parameters are consolidated in Table S3. The polymer is 150 chains of 20 beads ($\sigma_{pp}=1$) connected by finite-extensible nonlinear elastic (FENE) bonds (K=30, $R_{0}=1.5$); non-bonded polymer beads interact through a purely repulsive Weeks–Chandler–Andersen (WCA) potential. Fillers are 94 single beads of diameter $\sigma_{ff}=2$ at a filler volume fraction ϕ=0.20. Filler–filler interactions are attractive LJ ($\varepsilon_{ff}=2$, cutoff $2.5\sigma_{ff}$). The control variable is the polymer–filler well depth $\varepsilon_{pf}\in \left\{0.5,1.0,2.0\right\}$ (weak/medium/strong); a mobility proxy (thermostat temperature $T^{*}\in \left\{0.9,1.0,1.2\right\}$) is varied independently, giving a 3×3 campaign of 9 cells, augmented by three independent seeds at $T^{*}=1.0$ for a statistical uncertainty estimate. Each cell is prepared by overlap-removal minimization, a ramped soft push-off, and a constant-volume, constant-temperature (NVT) equilibration (verified by a potential-energy plateau, drift <0.3%, at the target temperature), then subjected to (a) a small-to-large oscillatory strain sweep via an oscillatory box tilt, and (b) a shear-cessation protocol (steady shear to rupture the network, then quiescent recovery). Equilibrium snapshots are used to count filler–filler contacts (center separation <2.5), polymer–filler contacts and the bound-polymer fraction (beads within 2.0 of a filler center), and the chain-bridging fraction (chains contacting ≥2 distinct fillers). The main grid holds ϕ=0.20 fixed to isolate the interaction effect; a separate loading sweep (ϕ∈{0.10, 0.20, 0.30} at fixed interaction) probes the network densification behind the $\gamma_{c}$ loading law. The oscillatory *modulus* from a system of this size is noisy and is therefore used only qualitatively; the *structural* descriptors, by contrast, are robust (Section 3.4).

### 2.5. Evaluation and Statistics

Generalization is assessed on three held-out axes of increasing difficulty. The main axis is leave-one-filler-class-out cross-validation: the model is trained on all but one filler chemistry and tested on the held-out one. Because the minimal model uses only the rubber family and loading—not filler chemistry—this axis measures how well the family-and-loading prior transfers to a chemistry excluded from training, rather than an explicit prediction of a new filler chemistry; the ablation of Section 3.8 confirms that adding the chemistry label does not improve it. Two harder axes stress the model further: leave-one-loading-out (holding out an entire filler loading) and leave-one-rubber-out (training on one rubber family and testing on the other). The mean and standard deviation of the mean absolute error (MAE) are reported over eight independent training seeds (the single-seed number is init-dependent and is not reported as a headline). Significance is tested with a paired Wilcoxon signed-rank test on per-sample absolute errors against each baseline, and a bootstrap 95% interval on the MAE is reported.

## 3. Results

### 3.1. Measured Total Softening Across the Benchmark

Figure 2 shows the normalized Payne-like curves. Active fillers (carbon black, silica) soften far more than nano-CaCO_3_, and BR–VMQ blends soften more than BR; the drop fraction reaches 0.79 (BR–VMQ–N220–30). Fitting Equation (1) to each compound reproduces the curve almost perfectly (median $R^{2}=0.997$); the fitted critical strain orders sensibly by filler activity (Table 1: silica $\gamma_{c}\approx 94\%$, carbon black ≈101%, CaCO_3_≈134%, unfilled ≈152%), i.e., more active fillers break at lower strain. But with per-sample parameters, Kraus has no predictive power for an unseen compound—it is a description, not a model.

**Figure 3:**
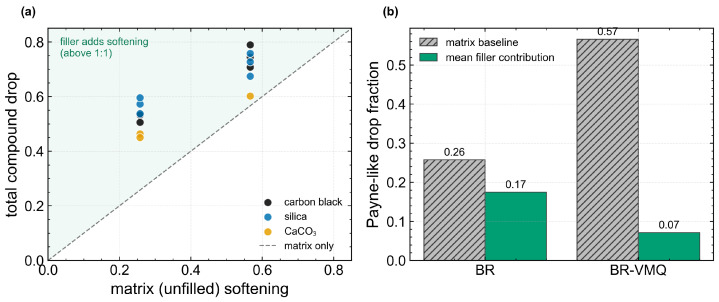
The unvulcanized compounds soften even without filler. (**a**): the total compound drop versus the unfilled-matrix baseline (at 30 phr). (**b**): the matrix baseline and the mean filler contribution per rubber—for BR–VMQ, the matrix dominates. The model targets the total (compounding-relevant) drop; the filler-network contribution (total minus baseline) still orders fillers correctly (carbon black ≈ silica > CaCO_3_).

A caveat is important: these compounds are unvulcanized, so even the unfilled references soften with strain (drop 0.26 for BR, 0.57 for BR–VMQ; Figure 3). The measured drop therefore mixes a raw-rubber matrix baseline with the filler-network contribution. In vulcanized filled rubbers, evidence points to deformation-induced breaking of the percolated filler network as the primary cause of the Payne effect, with the polymer matrix playing a secondary role [37]. Here, the matrix itself softens substantially, so we model the *total* measured drop—the compounding-relevant quantity—rather than an isolated filler-network term. The filler contribution (total minus the same-family unfilled baseline) is smaller (0.09 to 0.14 per filler class; Figure 3b resolves it by rubber, where the BR–VMQ filler term is much smaller than the matrix term ) and still orders the fillers correctly (carbon black ≈ silica > CaCO_3_). The balance is asymmetric between the two rubbers: for BR, the matrix baseline (0.26) is comparable to the filler-network increment, whereas for BR–VMQ, the matrix baseline (0.57) exceeds it by several folds, so the matrix-dominated description applies to the blend and not to BR.

### 3.2. Post-Fit Critical-Strain Scaling of the Measured Curves

**Figure 4:**
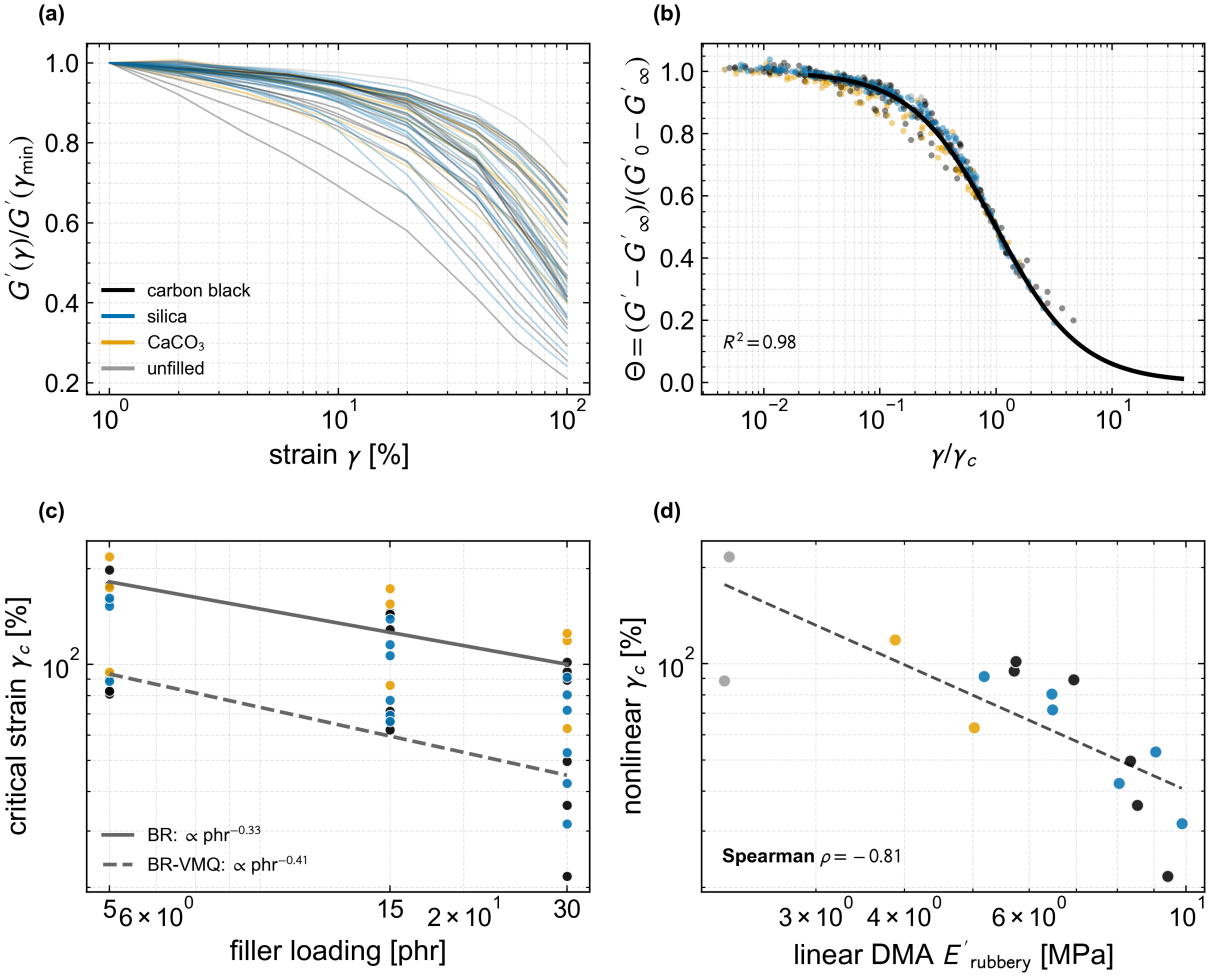
Post-fit critical-strain scaling of the measured total softening. (**a**) The 47 raw normalized curves; the roll-off strain spans 10×. (**b**) Rescaling by the per-compound fitted critical strain $\gamma_{c}$ gives a shared empirical shape $\Theta =1/(1+{\left(\gamma /\gamma_{c}\right)}^{2m})$, m=0.60, $R^{2}=0.98$. (**c**) The fitted $\gamma_{c}$ follows a power law in filler loading (BR solid, BR–VMQ dashed); at each loading the vertical spread reflects filler-class differences. (**d**) In the DMA subset (30 phr compounds and unfilled references), the linear dynamic mechanical analysis (DMA) rubbery modulus correlates with the fitted $\gamma_{c}$ (Spearman ρ=−0.81, n=16). Colors denote filler class as in Figure 2.

The 47 total-softening curves look diverse—the strain at which they roll off spans a tenfold range (Figure 4a). Much of this variation can be removed by a fitted critical strain. Fitting each curve to the Kraus form $G^{\prime }\left(\gamma \right)/G^{\prime }\left(\gamma_{\min}\right)=g_{\infty }+\left(1-g_{\infty }\right)/\left(1+{\left(\gamma /\gamma_{c}\right)}^{2m}\right)$ and rescaling the strain axis by the per-compound $\gamma_{c}$ gives a compact shared shape (Figure 4b): the normalized modulus $\Theta =\left(G^{\prime }-G_{\infty }^{\prime }\right)/\left(G_{0}^{\prime }-G_{\infty }^{\prime }\right)$ is described by a common function of $\gamma /\gamma_{c}$ with shape exponent m=0.60 and $R^{2}=0.98$ over all 517 measured points. This is a post-fit scaling result: $\gamma_{c}$ is extracted from the tested Payne-like curve, so the overlap by itself should not be read as an independent prediction for a new compound. Because the Kraus-type form already fits each individual curve very well, the overlap is best interpreted as a compression of fitted curve parameters and as a necessary precondition for composition-to-curve prediction, rather than as a standalone discovery. Shuffling $\gamma_{c}$ across compounds degrades the overlap ($R^{2}\;:0.98\;\to \;0.65$), and omitting the $\gamma_{c}$ rescaling fails outright ($R^{2}=-6.8$). The shared shape is approximate rather than exact—*m* narrows weakly with loading (0.68 to 0.54 across 5 to 30 phr), so allowing a per-compound *m* raises the overlap quality from $R^{2}=0.98$ to 0.997.

The Kraus form used here is a standard empirical description, and its breadth exponent is broadly consistent with the wider literature. Reported Kraus exponents for carbon-black and silica compounds typically lie around m≈0.5–0.7, with a residual dependence on filler type and loading; an independent study of eight carbon-black grades in natural rubber obtained unconstrained storage-modulus exponents averaging m≈0.44 and, for consistency across grades, adopted a fixed m=0.55 [38]. Our shared exponent (m=0.60) and the per-compound fits (m≈0.52–0.76 by filler class, Table 1) are of comparable order, so the same Kraus functional form describes an independent rubber and filler set. Only this dimensionless exponent is compared, since the absolute $\gamma_{c}$ is protocol- and modulus-dependent. This is not an external validation of the composition-to-curve prediction: no public butadiene-rubber Payne dataset with a formulation axis exists, so that prediction is assessed only on the internal held-out axes (Table 2).

**Figure 5:**
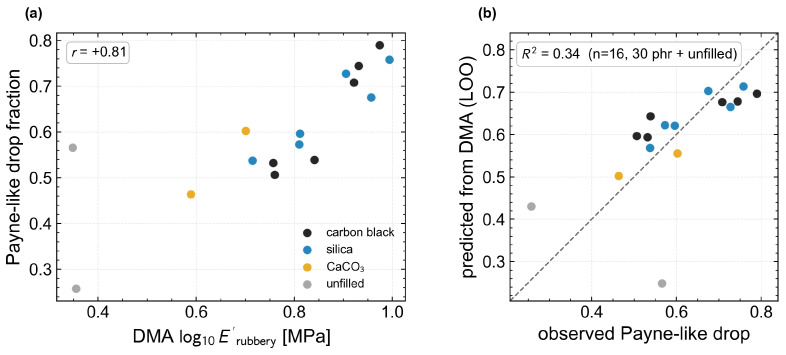
The linear DMA state also correlates with the measured softening magnitude in the DMA subset (30 phr compounds and unfilled references, n=16). (**a**): rubbery storage modulus versus Payne-like drop (Pearson r=+0.81 ). (**b**): the single-descriptor leave-one-out prediction ($R^{2}=0.34$). The modest leave-one-out $R^{2}$ reflects the small, single-loading subset (n=16) rather than a weak association: adding descriptors overfits and lowers the leave-one-out score, so this panel is reported as a correlation, not an optimized predictor—raising $R^{2}$ here would require more 30 phr compounds, not a different fit.

The compound specificity therefore reduces to a single number, the critical strain $\gamma_{c}$, and it carries clear physics. It falls as a power law in filler loading, $\gamma_{c}\propto {\mathrm{phr}}^{-0.33}$ (BR) and ${\mathrm{phr}}^{-0.41}$ (BR–VMQ) (Figure 4c), and the silicone-blend matrix yields at roughly half the strain of the butadiene matrix—a denser, more screened filler network breaks down earlier. The *linear* DMA state is also associated with the fitted nonlinear scaling strain: across the compounds with DMA data (the 30 phr compounds and the unfilled references), the rubbery storage modulus $E_{\mathrm{rubbery}}^{\prime }$ tracks $\gamma_{c}$ with Spearman ρ=−0.81 (Figure 4d). Within this DMA subset, this linear viscoelastic descriptor is informative about the fitted scaling strain, while the loading dependence is carried separately by the power law (Figure 4c). This is treated as a subset correlation, not as a standalone universal predictor. Equivalently, the same linear state correlates with the softening *magnitude*: across the 16 compounds with both DMA and Payne-like softening data the rubbery modulus correlates with the Payne-like drop at Pearson r=+0.81 (Figure 5). The two coefficients describe distinct quantities and are physically consistent—a stiffer rubbery network both yields at a lower scaling strain (Spearman ρ=−0.81 against $\gamma_{c}$) and softens more deeply (r=+0.81 against the drop). At this small, single-loading sample each is reported as a correlation, not a multivariate predictor (leave-one-out $R^{2}=0.34$).

### 3.3. Baselines: A Strain-Independent Spectrum Cannot Soften

**Figure 6:**
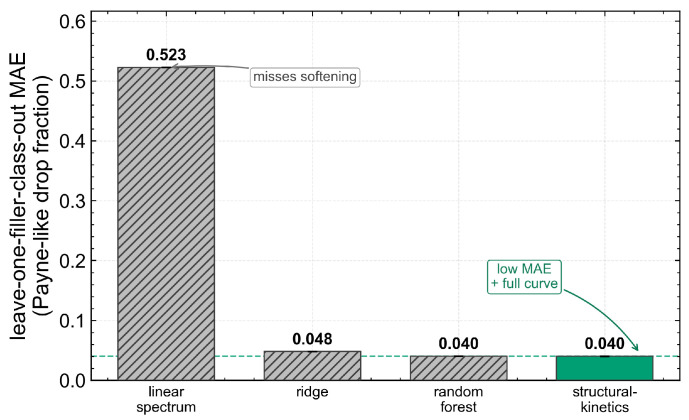
Leave-one-filler-class-out error on the Payne-like drop; all models use the rubber family and filler loading. An ablation tests whether adding filler-chemistry identity or MD descriptors gives incremental value in this dataset. The structural-kinetics model (0.040) ties the best scalar regressor—a random forest (p=0.87)—and is lower than the strain-independent linear spectrum, as expected for a model that explicitly contains amplitude softening. For the structural-kinetics model, the error bar denotes the standard deviation over eight training seeds. The green dashed line marks the structural-kinetics model’s MAE level for reference.

A linear viscoelastic spectrum predicts $G^{\prime }\left(\gamma \right)/G^{\prime }\left(\gamma_{\min}\right)=1$ at every strain; its error on the Payne-like drop is therefore the entire observed drop, with a mean absolute error MAE=0.52 (Figure 6, Table 3). Black-box regressors of the scalar drop from the rubber family and filler loading do far better—a ridge regression reaches a leave-one-filler-class-out MAE=0.048 and a random forest of 0.040. These frame the task: the physics is strongly nonlinear, and a useful model must reproduce the softening curve, not just its endpoint.

### 3.4. MD: Bound-Layer Screening and Filler Dispersion

**Figure 7:**
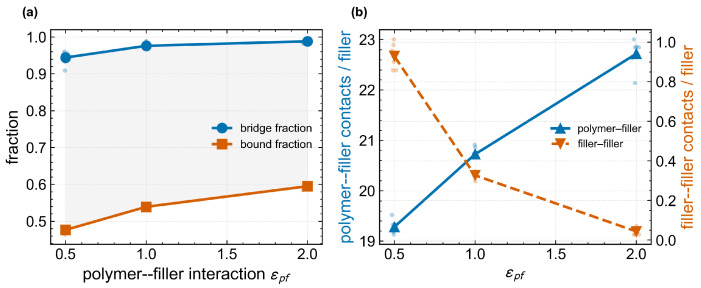
MD structural descriptors versus polymer–filler interaction $\varepsilon_{pf}$. (**a**): bound and bridge fractions. (**b**): polymer–filler (blue) and filler–filler (orange) contacts per filler. Bound-layer screening: bound fraction and polymer–filler contacts rise while direct filler–filler contacts vanish. Trends are robust across the three chain mobilities (Table 4).

**Figure 8:**
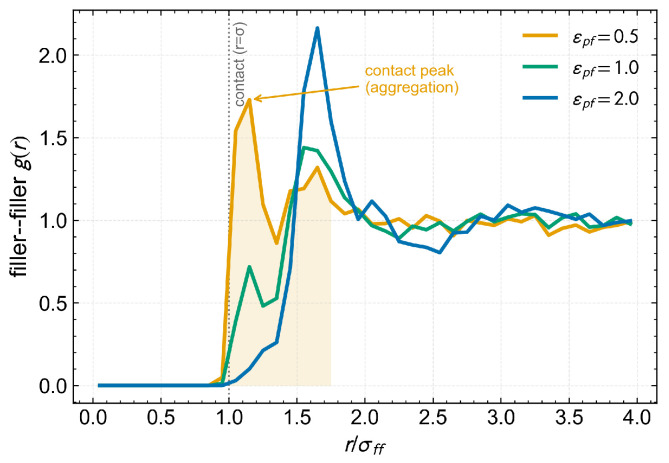
Filler–filler radial distribution function by interaction $\varepsilon_{pf}$: weak interaction gives a strong contact peak (aggregation); strong interaction disperses the fillers.

Figure 7 and Table 4 report the MD descriptors. As the polymer–filler interaction strengthens, the bound fraction rises (0.48→0.54→0.60) and polymer–filler contacts rise (19.4→22.7 per filler), while direct filler–filler contacts vanish (0.91→0.32→0.04): a thick bound layer coats the fillers and screens their direct contact. The ordering is monotonic, well separated, and robust both across the three chain mobilities and across three independent seeds (seed standard deviation ≤0.01 for the bound fraction and ≤0.08 for filler–filler contacts—far below the between-level differences; Table 4). The filler–filler radial distribution function (Figure 8) makes this structural: weak interaction produces a pronounced contact peak and the fillers aggregate into larger contacting clusters (largest cluster ∼10% of fillers, mean cluster size 1.75), whereas strong interaction disperses them into essentially isolated particles (mean cluster size 1.02; cluster statistics versus interaction in Figure S1). Even at the weakest interaction, the fillers do not percolate at ϕ=0.20 (largest cluster ∼10%), so this CG model captures the *bound-rubber and dispersion* contribution to reinforcement rather than a rigid percolating filler network.

### 3.5. MD Structure Under Strain: Bridges Rupture, the Bound Layer Holds

**Figure 9:**
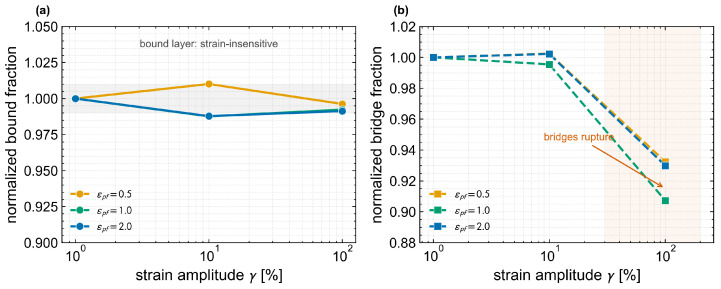
Molecular-dynamics (MD) structure during the oscillatory sweep. The bound layer is robust to strain ((**a**); change <1% up to 100% strain), whereas the bridging network ruptures at large amplitude ((**b**); bridge-fraction drop 7% to 9%)—a molecular analogue of Payne softening carried by bridge rupture.

Dumping the structure *during* the oscillatory sweep provides the molecular counterpart of the Payne-like curve (Figure 9). The bound layer is essentially strain-independent (it changes by <1% up to 100% strain): adsorbed chains do not desorb under oscillation at this scale. The *bridging* network, by contrast, ruptures at the largest amplitude (chain-bridging fraction drops 7% to 9% at 100% strain). The Payne-like softening in this MD scenario is therefore associated with rupture of polymer bridges between fillers, not bound-layer desorption—though the structural drop (<10%) is much smaller than the measured modulus drop (up to 0.8), a reminder that the CG model provides qualitative structural support rather than quantitative magnitude prediction. A shear-cessation protocol indicates that the network re-forms over time (the time-domain counterpart of the steady-state z(γ)), but at ϕ=0.20 the direct filler–filler contact count is too sparse (≲0.5 per filler) to quantify the recovery reliably; it is therefore reported only qualitatively.

### 3.6. The Structural-Kinetics Model: Full Curves, Learned Parameters

**Figure 10:**
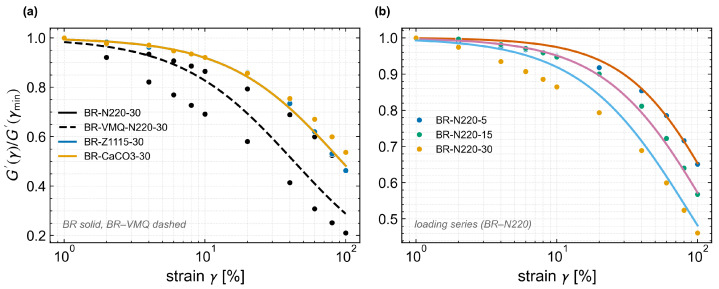
Structural-kinetics model (lines) against measured Payne-like softening data (points). (**a**): four representative compounds. (**b**): the BR–N220 loading series (5 to 30 phr). A single predicted parameter triple per compound breproduces the full curve (overall RMSE 0.031).

**Figure 11:**
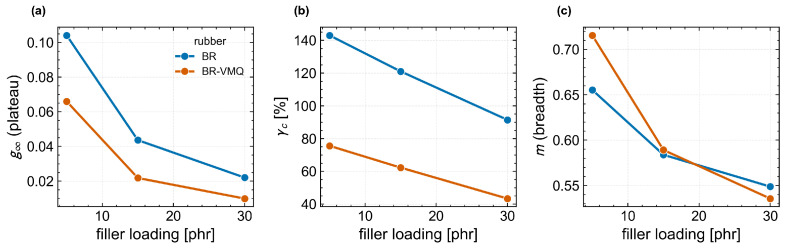
Learned structural parameters $\left(g_{\infty },\gamma_{c},m\right)$ versus filler loading for both rubbers (held-out predictions): (**a**) the residual-plateau fraction $g_{\infty }$; (**b**) the critical strain $\gamma_{c}$; (**c**) the breadth exponent *m*. All three decrease with loading: a denser filler network softens more deeply (smaller $g_{\infty }$) and yields at lower strain (smaller $\gamma_{c}$).

**Figure 12:**
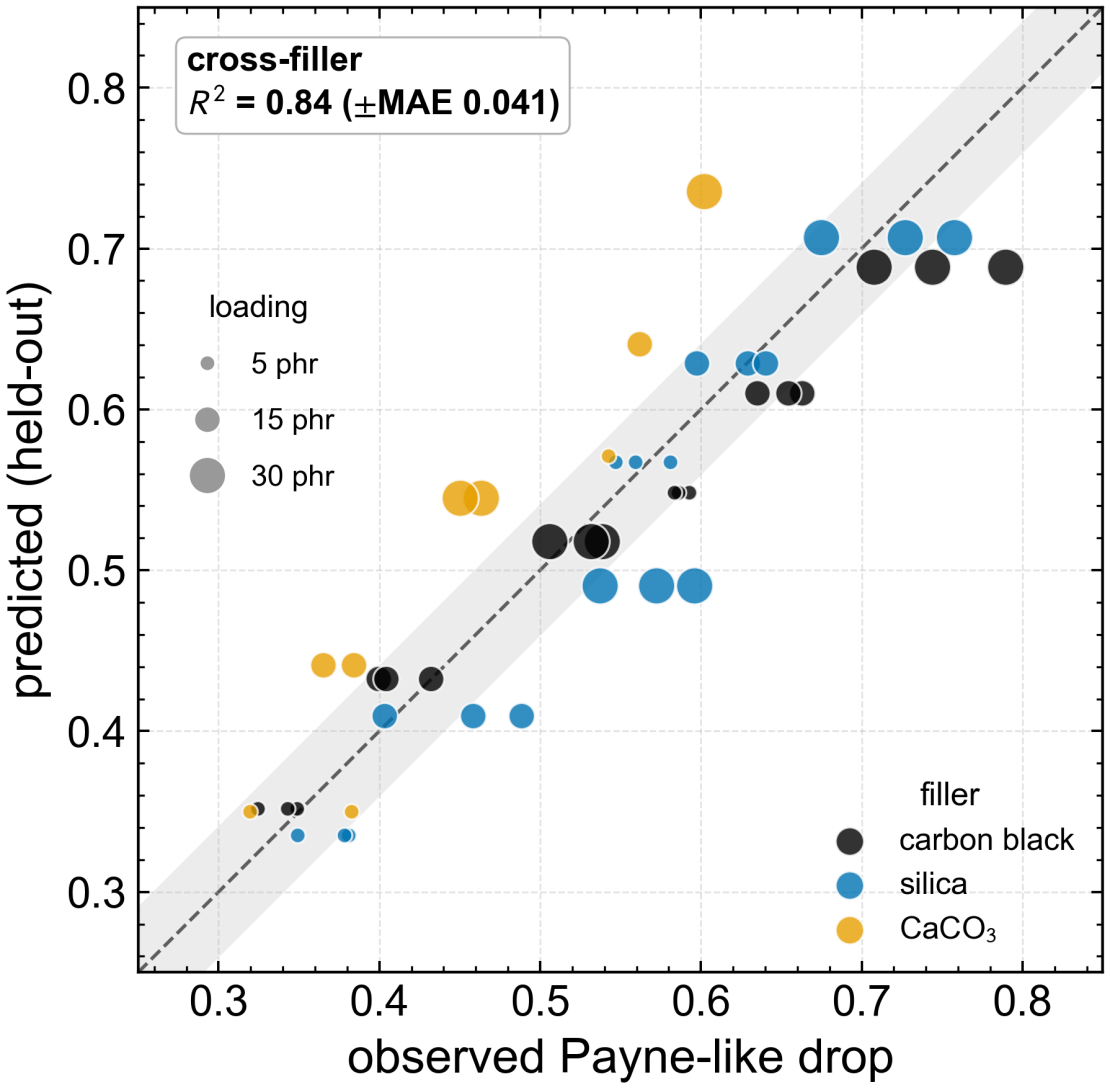
Held-out (leave-one-filler-class-out) predicted versus observed Payne-like drop fraction. Marker color encodes filler class and marker size encodes filler loading (5 to 30 phr); this makes the error structure visible—the largest residuals are the high-loading, weakly interacting nano-CaCO_3_ compounds, the hardest cases. Dashed line: identity; shaded band: ±MAE. The panel shows one training seed (cross-filler $R^{2}=0.84$); the headline drop MAE is the eight-seed mean, 0.040 (Table 3).

The model predicts the full normalized curve with a root-mean-square error of 0.031 (Figure 10): a single learned parameter triple—a function of the rubber family and filler loading—reproduces both rubbers across the loading series. Because the curves share the empirical shape of Section 3.2, this is equivalent to predicting the scaling parameter $\gamma_{c}$ from composition, which the model does at held-out Spearman r=0.89 (median error 19%), together with the residual plateau $g_{\infty }$. The learned parameters vary physically with loading (Figure 11): across 5 to 30 phr the residual plateau $g_{\infty }$ falls steeply (Spearman ρ=−0.82), the critical strain $\gamma_{c}$ drops from ∼143% to ∼91% for BR and from ∼76% to ∼43% for BR–VMQ (ρ=−0.47 pooled), and the breadth *m* narrows (ρ=−0.93)—a denser filler network leaves a smaller un-softenable core and yields at lower strain in both rubbers. These are physically named, interpretable quantities that a scalar regression never exposes. On the held-out cross-filler task the predicted and observed drop fractions track the identity line (Figure 12), with leave-one-filler-class-out MAE=0.040±0.000 over eight seeds. Encoding filler loading as marker size makes the residual structure plain: the largest errors are the high-loading, weakly interacting nano-CaCO_3_ compounds (held-out CaCO_3_ drop MAE=0.068, against 0.031 to 0.037 for the active carbon-black and silica fillers), consistent with loading being the model’s weakest axis (Table 2).

### 3.7. Statistical Validation

**Figure 13:**
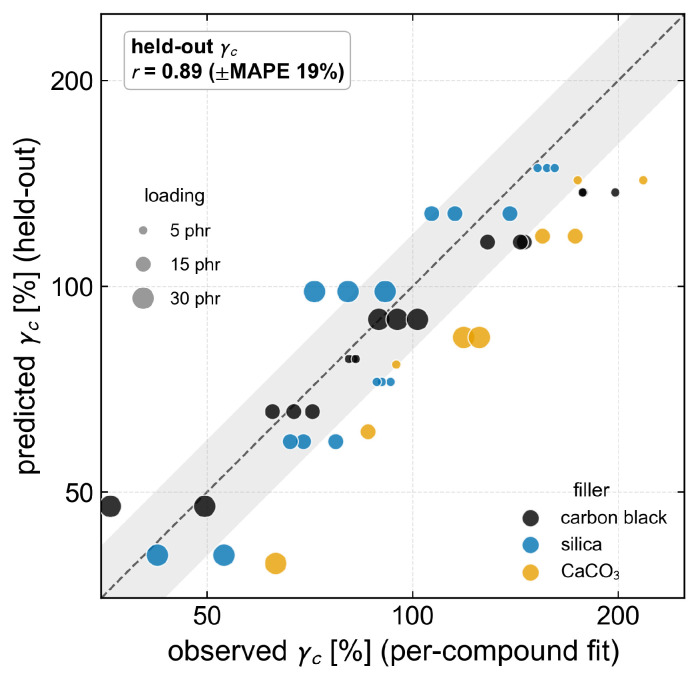
The model’s core task in the shared-shape framing: held-out (leave-one-filler-class-out) prediction of the scaling strain $\gamma_{c}$ against the per-compound fit (r=0.89 in $\log\gamma_{c}$; identity dashed, ±MAPE band). Marker color encodes filler class and size encodes loading (5 to 30 phr); the largest residuals are again the weakly interacting nano-CaCO_3_ compounds. A scalar drop regressor cannot return $\gamma_{c}$—the central scaling parameter in this shared-shape approximation.

The statistical comparisons are summarized in Figure 6 and Table 3. Relative to the linear spectrum, the error reduction is large and statistically significant (paired Wilcoxon $p=5.7\times 10^{-14}$). Against the black-box scalar regressors, the structural-kinetics model is statistically indistinguishable: it *ties* with the random forest (p=0.87) and the ridge regression (p=0.20) in drop accuracy—neither outperforming them nor being outperformed. This is an asymmetric but useful comparison: the random forest predicts only the scalar drop Δ, whereas the structural-kinetics model is trained on and returns the full normalized curve. The seed standard deviation rounds to 0.000 at the reported precision, but uncertainty is better represented by the bootstrap 95% interval [0.032,0.049]. As a stronger full-curve black-box control, a free-form MLP that predicts the eleven normalized curve points directly—with no structural-kinetics constraint, under the same rubber-family-and-loading features and the same leave-one-filler-class-out protocol with eight seeds—matches it on the Payne-like drop (MAE=0.040) and is in fact marginally better on the full-curve RMSE (0.030 versus 0.031; Table 3). The Kraus-type inductive bias therefore carries at most a negligible accuracy cost; it is an interpretability constraint—it yields the physically named parameters $\left(g_{\infty },\gamma_{c},m\right)$—rather than a source of superior accuracy. The constraint is not merely cosmetic: the Kraus form is monotonic and bounded by construction, so the predicted $G^{\prime }\left(\gamma \right)$ cannot take the non-physical, non-monotonic shapes that an unconstrained network can produce when extrapolated outside the measured window—a practical safeguard when the model is queried at an unseen composition. The model’s contribution is therefore what a scalar regressor cannot provide: the entire physically parameterized $G^{\prime }\left(\gamma \right)$ curve (RMSE 0.031) and a held-out prediction of the shared-shape scaling strain $\gamma_{c}$ at r=0.89 (Figure 13)—the central scaling parameter within the shared-shape approximation.

The central control is an *ablation*: does supplying filler-chemistry identity help at all? Table 2 compares the minimal model (rubber family and loading) against the same model given the filler-class label or the MD structural descriptors, on three held-out axes. On every axis the minimal model is best: adding the categorical label or the MD descriptors does not improve held-out prediction and slightly degrades it in this dataset—cross-rubber, the hardest axis, rises from 0.175 to 0.199. The MD descriptors are the worst on loading, because the fixed-ϕ MD carries no loading information, and they are 0.88-correlated with the categorical label: a continuous recoding of the same three classes, not new information. This pattern indicates that the Payne magnitude transfers through the rubber family and loading within the physical curve form, whereas explicit filler chemistry appears to overfit the seen chemistries and does not improve held-out generalization in this benchmark. Holding out an entire polymer matrix remains the hard axis (0.175), because the two rubbers differ systematically in their unfilled baseline (BR–VMQ softens far more even unfilled, Figure 3)—a rubber-level offset no filler feature can supply. As the most direct test of new-compound prediction, a leave-one-compound-out cross-validation—each of the 47 compounds held out singly and its curve predicted from the rubber family and loading—gives a Payne-like-drop MAE=0.038±0.001 over eight seeds, confirming that the family-and-loading prior transfers to a previously unseen individual compound, not only to a held-out chemistry class.

### 3.8. What the MD Contributes to Prediction

The ablation (Table 2) settles the role of the MD descriptors. As predictive features they do not help; being a continuous recoding of the three filler classes (correlation 0.88 with the categorical label), they essentially cannot. The MD is therefore not used as a predictive component of the final model. Its value is more limited: it provides qualitative structural context for an ordering already visible in the measurements. Stronger polymer–filler interaction thickens the bound layer and suppresses direct filler–filler contact (Figure 7 and Figure 8), and the amplitude sweep shows bridge rupture while the bound layer is nearly unchanged (Figure 9). This is consistent with the usual bound-layer-screening and bridge-rupture picture, but it is not an independent quantitative mechanism validation. On this benchmark, MD descriptors are not shown to add predictive information beyond the rubber family and loading.

**Figure 14:**
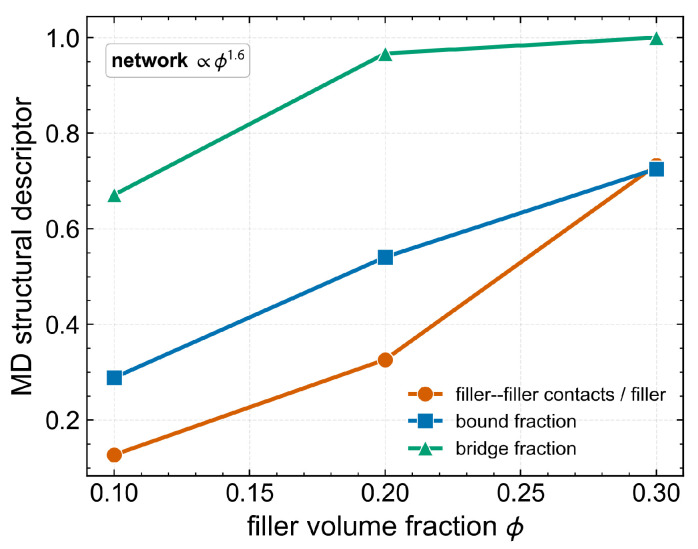
Molecular-dynamics (MD) loading sweep at fixed polymer–filler interaction (ϕ=0.10, 0.20 and 0.30). The filler–filler contact network, bound fraction and chain bridging rise steeply with filler volume fraction ϕ (contacts $\propto \phi^{1.6}$), a qualitative trend consistent with lower experimental $\gamma_{c}$ at higher filler loading (Figure 4c).

The volume-fraction sweep (Figure 14) gives a consistency check along the loading axis rather than a calibrated prediction. As ϕ increases from 0.10 to 0.30, contacts per filler rise as $\phi^{1.6}$, the bound fraction increases from 0.29 to 0.73 and the bridging fraction from 0.67 to 1.0. This trend is compatible with the experimental observation that higher loading yields lower $\gamma_{c}$ (Figure 4c), but the MD system is too coarse and too small to infer the experimental loading exponent directly.

## 4. Discussion

The central result is the composition-to-curve model, not the post-fit overlap alone. The overlap shows that the measured total softening curves can be compressed into a small set of Payne-like parameters; the predictive step is that these parameters can then be estimated from the rubber family and loading under held-out splits. Relative to classical Payne and filler-network models [1,6,10], the contribution is therefore not a new universal softening law but rather a benchmark-bound route from formulation descriptors to a full physically named curve. Relative to black-box scalar regression, the contribution is not a lower scalar-drop error; the model ties with the best scalar-drop predictor while returning an interpretable $G^{\prime }\left(\gamma \right)$ response.

The negative ablation result also needs this boundary. In this unvulcanized benchmark, the rubber family and loading carry most of the predictive signal for the total measured softening. This does not imply that filler chemistry is irrelevant to the Payne effect in general. Rather, explicit filler labels and the present MD descriptors do not add held-out predictive value once the dominant rubber-family and loading signals are included. The MD results are consequently used as qualitative structural context, not as a source of additional predictive features.

### Limitations

The shared scaling shape is empirical and post-fit: $\gamma_{c}$ is extracted from the tested curve for the overlap, so prediction of the overlap using only composition- or DMA-derived $\gamma_{c}$ would require separate validation. The shape is also not exact: the exponent narrows weakly with loading, and the power-law loading exponents are descriptive over three loading levels rather than independently established laws. The analysis rests on 47 compounds spanning two rubbers and three filler families—a clean but bounded diversity. An independent carbon-black/natural-rubber dataset reports a comparable Kraus exponent (Section 3.2), but a broader cross-supplier benchmark incorporating a formulation axis would still be needed to test the composition-to-curve step outside this benchmark.

The comparison with black-box models is made on both targets: beyond the scalar-drop random forest, a full-curve free-form MLP evaluated across the same held-out splits matches the structural-kinetics model on the entire curve (Table 3), so the Kraus-type constraint incurs no loss of accuracy and is retained purely for interpretability. The learned parameters are physically named because they inherit the Kraus form, but they are still predicted curve parameters rather than independently measured microstructural quantities.

The MD is a coarse-grained, methods-scale model: small systems, a 3×3 interaction–mobility campaign (seed-replicated only at one condition), and an oscillatory modulus too noisy at this size to use quantitatively (the analysis relies on the robust structural descriptors). The class-to-$\varepsilon_{pf}$ mapping defined in Section 2.4 is a physically motivated proxy, not a calibration; because ϕ is fixed by design, the MD descriptors omit most loading dependence—one reason they add no predictive value beyond the rubber family and loading.

Finally, the benchmark compounds are unvulcanized. The measured drop includes a raw-rubber matrix baseline (Figure 3) on top of the filler-network contribution, and the model deliberately targets this total measured softening. Therefore, the filler-label ablation should not be regarded as evidence that filler chemistry is generally unimportant; it shows only that filler chemistry is not resolved as an incremental predictor in this total-softening benchmark. A separated filler contribution, vulcanized compounds, direct recovery measurements, larger MD systems with a calibrated ϕ sweep, per-batch surface characterization, and complementary instrumented nanomechanical testing (for example, nanoindentation) of freshly prepared compounds would be needed to isolate the filler-network Payne term and to replace the class-to-$\varepsilon_{pf}$ proxy.

## 5. Conclusions

This study asked whether the total Payne-like softening curve of an unvulcanized filled rubber can be estimated from formulation descriptors while retaining physically named curve parameters. The results support a bounded composition-to-curve interpretation.

1. *Empirical curve compression.* Fitting a per-compound critical strain $\gamma_{c}$ brings the 47 measured total-softening curves onto a shared empirical shape ($R^{2}=0.98$). This is a post-fit compression of measured curves—not an independent prediction, and not a universal Payne law.
2. *Composition-to-curve prediction.* The structural-kinetics model—a machine-learned parameterization of a Kraus-type form, not a new constitutive law—estimates the full normalized curve from the rubber family and loading (RMSE 0.031) and ties the best scalar-drop predictor s(MAE 0.040; p=0.87 versus random forest, p=0.20 versus ridge). An unconstrained full-curve black-box baseline reaches comparable accuracy, so the Kraus form is an interpretability constraint rather than an accuracy advantage: the model’s value is the physically named, interpretable curve a scalar regressor cannot return. The prediction uses only the rubber family and loading and therefore does not claim to extrapolate to an unseen filler chemistry; the held-out errors grow on the harder loading and cross-rubber axes.
3. *Qualitative molecular-dynamics interpretation.* Explicit filler chemistry labels and the present MD-derived descriptors do not add held-out predictive value beyond the rubber family and loading in this dataset. The MD results remain useful as qualitative structural context for bound-layer screening and bridge rupture, but not as quantitative mechanism validation and not as a measurement of the internal variable.

The framework should therefore be read as an interpretable prediction model for total softening in this specific unvulcanized benchmark. Separating the filler-network Payne term from the raw-rubber matrix baseline, especially in vulcanized compounds, is the main requirement for extending the result beyond this scope.

## Figures and Tables

**Table 1:** Per-compound Kraus fit (Equation (1)) summarized by filler class: mean critical strain $\gamma_{c}$, exponent *m*, fit quality $R^{2}$.

Filler Class	*n*	$\gamma_{c}$ [%]	*m*	$R^{2}$
silica	18	93.7	0.66	0.998
carbon black	18	100.6	0.62	0.997
CaCO_3_	9	134.2	0.52	0.997
unfilled	2	152.2	0.76	0.997

**Table 2:** Filler-representation ablation: leave-one-axis-out MAE (mean ± s.d. over six seeds). On every axis, adding filler-chemistry identity—a categorical label or the MD structural descriptors—does *not* show an improvement over rubber family + loading alone on this dataset. Bold marks the lowest MAE on each held-out axis.

Held-Out Axis	Family + Loading	+ Filler Label	+ MD Descriptors
filler chemistry	0.040±0.000	0.049	0.049
loading (phr)	0.059±0.002	0.060	0.072
polymer matrix (BR↔BR–VMQ)	0.175±0.018	0.199	0.199

**Table 3:** Cross-filler prediction of the Payne-like drop (leave-one-filler-class-out). All predictors use the rubber family and filler loading; the filler-chemistry ablation is examined separately. The free-form MLP is an unconstrained, full-curve black-box baseline (no Kraus form): it matches the interpretable structural-kinetics model in both the scalar drop and full-curve RMSE, indicating that the Kraus constraint incurs no loss of accuracy. Errors are reported as mean ± s.d. over eight training seeds where applicable. The paired significance tests are computed on the 45 filler-bearing compounds (the two unfilled references carry no filler class), so the baseline errors used for the *p*-values differ marginally from the tabulated leave-one-filler-class-out MAEs. Bold marks the structural-kinetics model proposed in this work; the italicized rows report the paired significance tests.

Model	MAE	Note
linear viscoelastic spectrum	0.52	no amplitude softening
ridge (family + loading)	0.048	scalar drop only
random forest (family + loading)	0.040	scalar drop only
free-form MLP (family + loading)	0.040±0.000	full curve, RMSE 0.030
**structural-kinetics**	0.040±0.000	full curve, RMSE 0.031
*significance (paired Wilcoxon, per-sample |error|):*		
vs. linear spectrum	$p=5.7\times 10^{-14}$ (large difference)	
vs. random forest	p=0.87 (not significant; a tie)	
vs. ridge regression	p=0.20 (not significant)	
bootstrap 95% CI (MAE)	[0.032,0.049]	
leave-one-loading-out	MAE 0.059 (harder axis)	

**Table 4:** MD structural descriptors for all 9 cells (interaction $\varepsilon_{pf}\times$ mobility $T^{*}$). Bound-layer screening is monotonic in $\varepsilon_{pf}$ and robust across mobility and across three independent seeds (seed s.d. ≤0.01 bound fraction, ≤0.08 filler–filler contacts).

$\varepsilon_{\mathrm{pf}}$	$T^{*}$	Bound Frac.	p–f Contacts	f–f Contacts	Bridge Frac.
0.5	0.9	0.481	19.52	0.957	0.951
0.5	1.0	0.483	19.33	0.858	0.956
0.5	1.2	0.477	19.21	0.901	0.933
1.0	0.9	0.548	20.91	0.326	0.987
1.0	1.0	0.541	20.74	0.326	0.967
1.0	1.2	0.531	20.36	0.298	0.989
2.0	0.9	0.604	23.00	0.028	0.984
2.0	1.0	0.599	22.84	0.028	0.989
2.0	1.2	0.587	22.14	0.050	0.991

## Data Availability

The public benchmark is the 4TU.ResearchData dataset (Ref. [30], CC BY 4.0). The processed data tables, validation split definitions, random seeds, analysis scripts, molecular-dynamics input templates, statistical validation scripts, and figure-generation scripts are available from the corresponding author upon reasonable request and will be deposited in a public repository upon acceptance.

## Supplementary Materials

The following supporting information can be downloaded at https://www.mdpi.com/article/10.3390/polym18141761/s1, Table S1: Per-compound Kraus parameters for all 47 benchmark compounds; Table S2: Held-out MAE on the Payne-like drop, resolved per filler class (leave-one-filler-class-out cross-validation); Table S3: Coarse-grained molecular-dynamics simulation parameters; Figure S1: Filler-cluster structure as a function of the polymer–filler interaction $\varepsilon_{pf}$; Figure S2: The learned residual-plateau parameter $1-g_{\infty }$ correlates with the observed Payne-like drop across compounds (r=0.65, n=45); Figure S3: Filler–filler contacts per filler after shear cessation, aggregated over the cells at each interaction strength (mean ± standard deviation).

## References

[1] Leblanc J. (2002). Rubber-filler interactions and rheological properties in filled compounds. Prog. Polym. Sci..

[2] Heinrich G., Klüppel M., Vilgis T.A. (2002). Reinforcement of elastomers. Curr. Opin. Solid State Mater. Sci..

[3] Robertson C.G., Hardman N.J. (2021). Nature of Carbon Black Reinforcement of Rubber: Perspective on the Original Polymer Nanocomposite. Polymers.

[4] Payne A.R. (1962). The Dynamic Properties of Carbon Black-Loaded Natural Rubber Vulcanizates. Part I. J. Appl. Polym. Sci..

[5] Fletcher W.P., Gent A.N. (1954). Nonlinearity in the Dynamic Properties of Vulcanized Rubber Compounds. Rubber Chem. Technol..

[6] Wang M.J. (1998). Effect of Polymer-Filler and Filler-Filler Interactions on Dynamic Properties of Filled Vulcanizates. Rubber Chem. Technol..

[7] Medalia A.I. (1972). Effective Degree of Immobilization of Rubber Occluded within Carbon Black Aggregates. Rubber Chem. Technol..

[8] Montes H., Lequeux F., Berriot J. (2003). Influence of the Glass Transition Temperature Gradient on the Nonlinear Viscoelastic Behavior in Reinforced Elastomers. Macromolecules.

[9] Stöckelhuber K.W., Das A., Jurk R., Heinrich G. (2010). Contribution of physico-chemical properties of interfaces on dispersibility, adhesion and flocculation of filler particles in rubber. Polymer.

[10] Klüppel M. (2003). The Role of Disorder in Filler Reinforcement of Elastomers on Various Length Scales. Adv. Polym. Sci..

[11] Heinrich G., Klüppel M. (2002). Recent Advances in the Theory of Filler Networking in Elastomers. Adv. Polym. Sci..

[12] Baeza G.P., Genix A.C., Degrandcourt C., Petitjean L., Gummel J., Couty M., Oberdisse J. (2013). Multiscale Filler Structure in Simplified Industrial Nanocomposite Silica/SBR Systems Studied by SAXS and TEM. Macromolecules.

[13] Domurath J., Saphiannikova M., Ausias G., Heinrich G. (2012). Modelling of stress and strain amplification effects in filled polymer melts. J. Non-Newton. Fluid Mech..

[14] Honerkamp J., Weese J. (1989). Determination of the relaxation spectrum by a regularization method. Macromolecules.

[15] Raissi M., Perdikaris P., Karniadakis G. (2019). Physics-informed neural networks: A deep learning framework for solving forward and inverse problems involving nonlinear partial differential equations. J. Comput. Phys..

[16] Karniadakis G.E., Kevrekidis I.G., Lu L., Perdikaris P., Wang S., Yang L. (2021). Physics-informed machine learning. Nat. Rev. Phys..

[17] Mahmoudabadbozchelou M., Jamali S. (2021). Rheology-Informed Neural Networks (RhINNs) for Forward and Inverse Metamodelling of Complex Fluids. Sci. Rep..

[18] Saadat M., Mahmoudabadbozchelou M., Jamali S. (2022). Data-Driven Selection of Constitutive Models via Rheology-Informed Neural Networks. Rheol. Acta.

[19] Lennon K.R., McKinley G.H., Swan J.W. (2023). Scientific machine learning for modeling and simulating complex fluids. Proc. Natl. Acad. Sci. USA.

[20] Linka K., Kuhl E. (2023). A new family of Constitutive Artificial Neural Networks towards automated model discovery. Comput. Methods Appl. Mech. Eng..

[21] Linden L., Klein D.K., Kalina K.A., Brummund J., Weeger O., Kästner M. (2023). Neural networks meet hyperelasticity: A guide to enforcing physics. J. Mech. Phys. Solids.

[22] Abdolazizi K.P., Linka K., Cyron C.J. (2024). Viscoelastic constitutive artificial neural networks (vCANNs)—A framework for data-driven anisotropic nonlinear finite viscoelasticity. J. Comput. Phys..

[23] Masi F., Stefanou I., Vannucci P., Maffi-Berthier V. (2021). Thermodynamics-based Artificial Neural Networks for constitutive modeling. J. Mech. Phys. Solids.

[24] Taç V., Rausch M.K., Sahli Costabal F., Buganza Tepole A. (2023). Data-driven anisotropic finite viscoelasticity using neural ordinary differential equations. Comput. Methods Appl. Mech. Eng..

[25] Malashin I., Tynchenko V., Gantimurov A., Nelyub V., Borodulin A. (2025). Physics-Informed Neural Networks in Polymers: A Review. Polymers.

[26] Deng W., Zhao Y., Zheng Y., Yin Y., Huan Y., Liu L., Wang D. (2024). Machine learning assisted analysis and prediction of rubber formulation using existing databases. Artif. Intell. Chem..

[27] Uruk Z., Kiraz A., Deniz V. (2022). A comparison of machine learning methods to predict rheometric properties of rubber compounds. J. Rubber Res..

[28] Kojima T., Washio T., Hara S., Koishi M. (2020). Synthesis of computer simulation and machine learning for achieving the best material properties of filled rubber. Sci. Rep..

[29] Madera Sabik R.C., Lejeunes S., Euchler E. (2025). Modeling the Payne effect in filled rubbers: Accounting for time effect and partial reversibility. Polym. Bull..

[30] Anyszka R., Blume A., Jia L. (2023). Data Underlying the Publication: Tires for Mars Rovers: Reinforcing BR and BR/Vinyl-Methyl Silicone Rubber Compounds with Carbon Black, Nano-CaCO_3_, or Silica for Good Low-Temperature Dynamic-Mechanical Performance.

[31] Anyszka R., Jia L., Blume A. (2024). Tires for Mars Rovers: Reinforcing BR and BR/Vinyl-Methyl Silicone Rubber Compounds with Carbon Black, Nano-CaCO3, or Silica for Good Low-Temperature Dynamic-Mechanical Performance. Tire Sci. Technol..

[32] Vogiatzis G.G., Theodorou D.N. (2018). Multiscale Molecular Simulations of Polymer-Matrix Nanocomposites. Arch. Comput. Methods Eng..

[33] Hagita K., Morita H., Takano H. (2016). Molecular dynamics simulation study of a fracture of filler-filled polymer nanocomposites. Polymer.

[34] Kremer K., Grest G.S. (1990). Dynamics of Entangled Linear Polymer Melts: A Molecular-Dynamics Simulation. J. Chem. Phys..

[35] Plimpton S. (1995). Fast Parallel Algorithms for Short-Range Molecular Dynamics. J. Comput. Phys..

[36] Thompson A.P., Aktulga H.M., Berger R., Bolintineanu D.S., Brown W.M., Crozier P.S., in ’t Veld P.J., Kohlmeyer A., Moore S.G., Nguyen T.D. (2022). LAMMPS—A flexible simulation tool for particle-based materials modeling at the atomic, meso, and continuum scales. Comput. Phys. Commun..

[37] Warasitthinon N., Genix A.C., Sztucki M., Oberdisse J., Robertson C.G. (2019). The Payne Effect: Primarily Polymer-Related or Filler-Related Phenomenon?. Rubber Chem. Technol..

[38] Rutherford K.J., Akutagawa K., Ramier J.L., Tunnicliffe L.B., Busfield J.J.C. (2023). The Influence of Carbon Black Colloidal Properties on the Parameters of the Kraus Model. Polymers.

