# Supplementary material for: Interpretable Composition-to-Curve Prediction of Payne-like Softening in an Unvulcanized BR/BR–VMQ Benchmark: Critical-Strain Scaling and Qualitative Molecular-Dynamics Context
Source: Polymers (Basel). 2026 Jul 18;18(14):1761. doi: 10.3390/polym18141761 (PMC13417734; doi:10.3390/polym18141761)
Supplement: Supplementary file 1 [file polymers-18-01761-s001.zip › polymers-4394005-supplementary.pdf]

# Supplementary Materials

## Interpretable Composition-to-Curve Prediction of Payne-like Softening in an Unvulcanized BR/BR–VMQ Benchmark: Critical-Strain Scaling and Qualitative Molecular-Dynamics Context

Yancai Sun, Feng Shi, Jian Xu, Wenjuan Bai,
Dianming Chu, Peiwu Hou and Wenzhong Deng

## S1 Per-compound critical-strain fits

Table 1 of the main text summarizes the Kraus fits by filler class. Table S1 gives the underlying per-compound parameters for each of the **47** benchmark compounds: the critical strain $\gamma_c$, breadth exponent $m$, Payne-like drop $\Delta = (G'_0 - G'_\infty)/G'_0$, normalized residual plateau $g_\infty = G'_\infty/G'_0$, and fit $R^2$. The fits are uniformly good (median $R^2 = 0.997$, minimum $R^2 = 0.991$), but the parameters are extracted *per compound* and carry no cross-compound predictive content on their own.

Table S1: Per-compound Kraus parameters for all 47 benchmark compounds. phr, parts per hundred rubber; $\gamma_c$ in %.

| Compound | Family | Filler | phr | $\gamma_c$ (%) | $m$ | $\Delta$ | $g_\infty$ | $R^2$ |
|---|---|---|---|---|---|---|---|---|
| BR–CaCO3–5 | BR | nano-$CaCO_3$ | 5 | 174.5 | 0.485 | 0.383 | 0.000 | 0.997 |
| BR–CaCO3NoCA–5 | BR | nano-$CaCO_3$ | 5 | 217.6 | 0.523 | 0.320 | 0.000 | 0.996 |
| BR–CaCO3–15 | BR | nano-$CaCO_3$ | 15 | 154.9 | 0.580 | 0.384 | 0.000 | 0.999 |
| BR–CaCO3NoCA–15 | BR | nano-$CaCO_3$ | 15 | 173.0 | 0.516 | 0.365 | 0.000 | 0.998 |
| BR–CaCO3–30 | BR | nano-$CaCO_3$ | 30 | 118.8 | 0.509 | 0.463 | 0.000 | 0.999 |
| BR–CaCO3NoCA–30 | BR | nano-$CaCO_3$ | 30 | 125.2 | 0.481 | 0.450 | 0.000 | 0.999 |
| BR–N220–5 | BR | carbon black | 5 | 178.0 | 0.600 | 0.349 | 0.000 | 0.996 |
| BR–N330–5 | BR | carbon black | 5 | 198.0 | 0.588 | 0.325 | 0.000 | 0.995 |
| BR–N550–5 | BR | carbon black | 5 | 177.4 | 0.631 | 0.343 | 0.000 | 0.996 |
| BR–N220–15 | BR | carbon black | 15 | 128.7 | 0.614 | 0.432 | 0.000 | 0.999 |
| BR–N330–15 | BR | carbon black | 15 | 145.6 | 0.636 | 0.400 | 0.000 | 0.997 |
| BR–N550–15 | BR | carbon black | 15 | 143.8 | 0.610 | 0.404 | 0.000 | 0.997 |
| BR–N220–30 | BR | carbon black | 30 | 89.2 | 0.432 | 0.539 | 0.000 | 0.996 |
| BR–N330–30 | BR | carbon black | 30 | 95.0 | 0.558 | 0.532 | 0.000 | 0.997 |
| BR–N550–30 | BR | carbon black | 30 | 101.7 | 0.605 | 0.506 | 0.000 | 0.999 |
| BR–REF | BR | unfilled | 0 | 215.9 | 0.721 | 0.257 | 0.000 | 0.997 |
| BR–U7000–5 | BR | silica | 5 | 157.2 | 0.650 | 0.381 | 0.000 | 0.993 |
| BR–U9100–5 | BR | silica | 5 | 152.4 | 0.681 | 0.378 | 0.000 | 0.996 |
| BR–Z1115–5 | BR | silica | 5 | 161.4 | 0.699 | 0.349 | 0.000 | 0.998 |
| BR–U7000–15 | BR | silica | 15 | 115.3 | 0.696 | 0.458 | 0.000 | 0.999 |
| BR–U9100–15 | BR | silica | 15 | 106.6 | 0.687 | 0.489 | 0.000 | 0.999 |
| BR–Z1115–15 | BR | silica | 15 | 138.8 | 0.636 | 0.403 | 0.000 | 0.999 |

*Table S1 continued*

| Compound | Family | Filler | phr | $\gamma_c$ (%) | $m$ | $\Delta$ | $g_\infty$ | $R^2$ |
|---|---|---|---|---|---|---|---|---|
| BR–U7000–30 | BR | silica | 30 | 80.4 | 0.608 | 0.572 | 0.000 | 0.999 |
| BR–U9100–30 | BR | silica | 30 | 71.8 | 0.578 | 0.596 | 0.000 | 0.999 |
| BR–Z1115–30 | BR | silica | 30 | 91.1 | 0.616 | 0.537 | 0.000 | 0.999 |
| BR–VMQ–CaCO3–5 | BR–VMQ | nano-$CaCO_3$ | 5 | 94.6 | 0.775 | 0.543 | 0.000 | 0.995 |
| BR–VMQ–CaCO3–15 | BR–VMQ | nano-$CaCO_3$ | 15 | 86.0 | 0.439 | 0.562 | 0.000 | 0.993 |
| BR–VMQ–CaCO3–30 | BR–VMQ | nano-$CaCO_3$ | 30 | 63.0 | 0.407 | 0.602 | 0.000 | 0.997 |
| BR–VMQ–N220–5 | BR–VMQ | carbon black | 5 | 81.9 | 0.762 | 0.593 | 0.000 | 0.996 |
| BR–VMQ–N330–5 | BR–VMQ | carbon black | 5 | 80.7 | 0.773 | 0.586 | 0.000 | 0.999 |
| BR–VMQ–N550–5 | BR–VMQ | carbon black | 5 | 82.6 | 0.794 | 0.584 | 0.000 | 0.999 |
| BR–VMQ–N220–15 | BR–VMQ | carbon black | 15 | 62.3 | 0.615 | 0.663 | 0.000 | 0.996 |
| BR–VMQ–N330–15 | BR–VMQ | carbon black | 15 | 67.0 | 0.665 | 0.654 | 0.000 | 0.995 |
| BR–VMQ–N550–15 | BR–VMQ | carbon black | 15 | 71.3 | 0.728 | 0.635 | 0.000 | 0.998 |
| BR–VMQ–N220–30 | BR–VMQ | carbon black | 30 | 21.7 | 0.417 | 0.790 | 0.000 | 0.995 |
| BR–VMQ–N330–30 | BR–VMQ | carbon black | 30 | 36.1 | 0.482 | 0.744 | 0.000 | 0.995 |
| BR–VMQ–N550–30 | BR–VMQ | carbon black | 30 | 49.6 | 0.579 | 0.708 | 0.000 | 0.996 |
| BR–VMQ–REF | BR–VMQ | unfilled | 0 | 88.5 | 0.789 | 0.566 | 0.000 | 0.996 |
| BR–VMQ–U7000–5 | BR–VMQ | silica | 5 | 92.9 | 0.761 | 0.547 | 0.000 | 0.997 |
| BR–VMQ–U9100–5 | BR–VMQ | silica | 5 | 90.1 | 0.720 | 0.559 | 0.000 | 0.996 |
| BR–VMQ–Z1115–5 | BR–VMQ | silica | 5 | 88.6 | 0.756 | 0.581 | 0.000 | 0.991 |
| BR–VMQ–U7000–15 | BR–VMQ | silica | 15 | 69.2 | 0.643 | 0.629 | 0.000 | 0.998 |
| BR–VMQ–U9100–15 | BR–VMQ | silica | 15 | 66.2 | 0.665 | 0.640 | 0.000 | 0.999 |
| BR–VMQ–Z1115–15 | BR–VMQ | silica | 15 | 77.2 | 0.668 | 0.598 | 0.000 | 0.999 |
| BR–VMQ–U7000–30 | BR–VMQ | silica | 30 | 42.3 | 0.584 | 0.727 | 0.000 | 1.000 |
| BR–VMQ–U9100–30 | BR–VMQ | silica | 30 | 31.6 | 0.628 | 0.758 | 0.061 | 1.000 |
| BR–VMQ–Z1115–30 | BR–VMQ | silica | 30 | 52.9 | 0.547 | 0.675 | 0.000 | 0.999 |

# S2 Held-out error by filler class and loading

Table 2 of the main text reports the overall held-out error of each predictor. Table S2 resolves it by filler class: $CaCO_3$ is the most challenging chemistry for every predictor, whereas the active fillers (carbon black and silica) are easier. On the separate leave-one-loading-out axis (whose overall value is reported in Table 4 of the main text), the per-loading errors are 0 phr, 0.168; 5 phr, 0.059; 15 phr, 0.038; and 30 phr, 0.068; the unfilled limit dominates the error. Replacing the physically named curve head with an unconstrained free-form head (using the same features and protocol) gives a statistically equivalent error—a Payne-like-drop MAE of 0.040 and a full-curve RMSE of 0.030, versus 0.040 and 0.031 for the Kraus-form head—so the physical parameterization is obtained at no cost in accuracy, only added interpretability.

Table S2: Held-out MAE on the Payne-like drop, resolved per filler class (leave-one-filler-class-out cross-validation).

| Predictor | Carbon black | Silica | $CaCO_3$ |
|---|---|---|---|
| Ridge regression | 0.041 | 0.040 | 0.064 |
| Random forest | 0.028 | 0.039 | 0.066 |
| Structural-kinetics model | 0.031 | 0.037 | 0.068 |

# S3 Coarse-grained molecular-dynamics parameters

Table S3 consolidates the Kremer–Grest bead–spring parameters described in the Methods section (§2.4); all quantities are in Lennard-Jones (LJ) reduced units. The resulting per-cell structural descriptors are shown in Table 3 of the main text.

Table S3: Coarse-grained molecular-dynamics simulation parameters.

| Quantity | Value |
|---|---|
| Engine | LAMMPS |
| Units | Lennard-Jones (reduced) |
| Polymer chains | 150 chains × 20 beads |
| Polymer bead diameter $\sigma_{pp}$ | 1 |
| Bonds | FENE ($K = 30$, $R_0 = 1.5$) |
| Non-bonded polymer | WCA (purely repulsive) |
| Filler beads | 94 single beads |
| Filler diameter $\sigma_{ff}$ | 2 |
| Filler volume fraction $\phi$ | 0.20 (fixed on the main grid) |
| Filler–filler interaction | attractive LJ, $\varepsilon_{ff} = 2$, cutoff $2.5\,\sigma_{ff}$ |
| Polymer–filler well depth $\varepsilon_{pf}$ | {0.5, 1.0, 2.0} (weak/medium/strong) |
| Mobility proxy temperature $T^*$ | {0.9, 1.0, 1.2} |
| Campaign | 3 × 3 grid (9 cells) + 3 seeds at $T^* = 1.0$ |
| Loading sweep (separate) | $\phi \in \{0.10, 0.20, 0.30\}$ |
| Bound-layer cutoff | 2.0 from a filler center |
| Filler–filler contact cutoff | 2.5 (center separation) |
| Bridging chain | contacts ≥ 2 distinct fillers |
| Protocols | (i) oscillatory strain sweep; (ii) shear cessation + recovery |

# S4 Additional molecular-dynamics structural results

The oscillatory modulus from a system of this size is noisy and is used only qualitatively; the structural descriptors below are robust and reproduce the interaction-strength ordering reported in the main text. Figure S1 shows the filler-cluster structure as a function of the polymer–filler interaction; Figure S2 shows that the model's internal softening variable $1 - g_\infty$ tracks the observed Payne-like drop ($r = 0.65$, $n = 45$); and Figure S3 reports the filler–filler contact level after shear cessation.

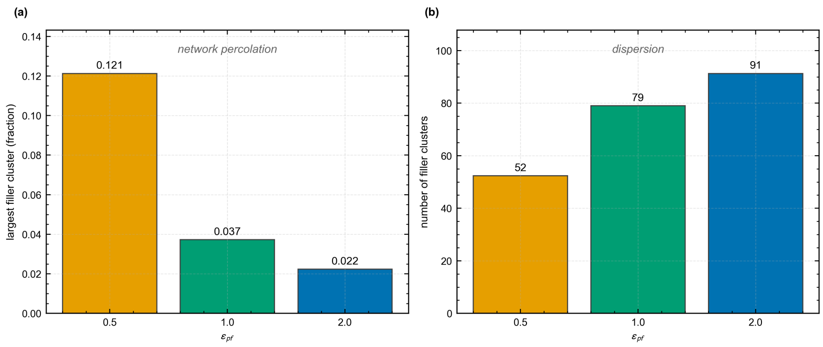


Figure S1: Filler-cluster structure as a function of the polymer–filler interaction $\varepsilon_{pf}$. (**a**) The largest filler cluster shrinks from a percolating fraction at weak interaction to a dispersed state at strong interaction; (**b**) the number of distinct clusters rises correspondingly. Stronger polymer–filler attraction screens filler–filler contacts and disperses the network.

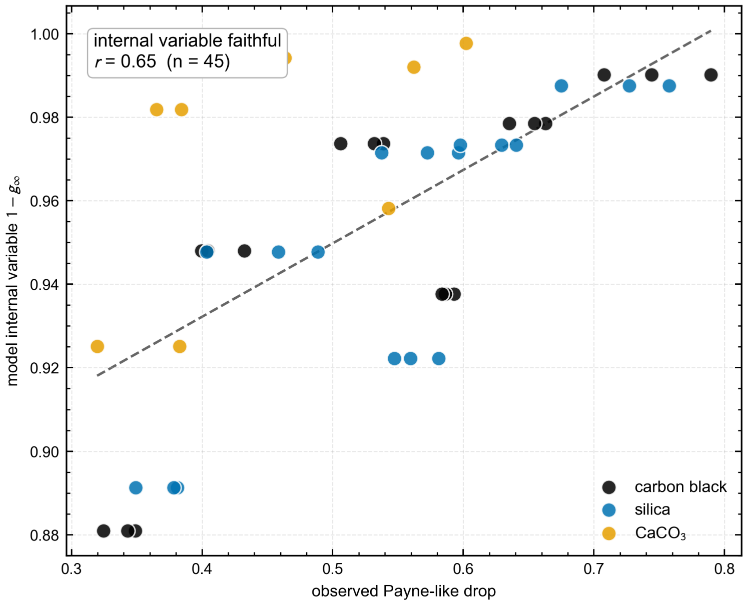


Figure S2: The learned residual-plateau parameter $1 - g_\infty$ correlates with the observed Payne-like drop across compounds ($r = 0.65$, $n = 45$). This is a consistency check on a predicted curve parameter; as stated in the main text, the internal variable is phenomenological, not an independently measured microstructural quantity.

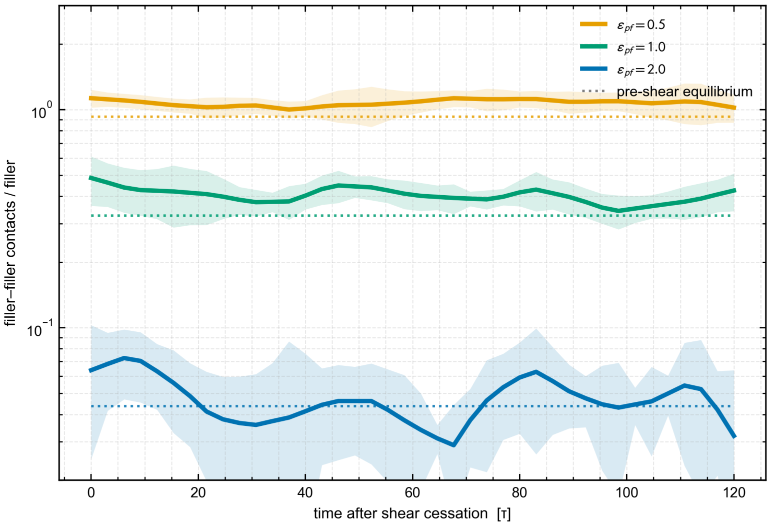


Figure S3: Filler–filler contacts per filler after shear cessation, aggregated over the cells at each interaction strength (mean ± standard deviation). The dispersion level is set by $\varepsilon_{pf}$ and stays near the pre-shear equilibrium (dotted lines); the contact count is too sparse at this volume fraction to resolve a slow re-aggregation transient, so recovery is reported only qualitatively.
